# Health-related quality of life using WHODAS 2.0 and associated factors 1 year after stroke in Korea: a multi-centre and cross-sectional study

**DOI:** 10.1186/s12883-022-03032-2

**Published:** 2022-12-24

**Authors:** Hey Jean Lee, Jung-Kook Song, Jiyoung Moon, Keonyeop Kim, Hyeung-Keun Park, Gil-Won Kang, Jun-Ho Shin, Jongsoo Kang, Byoung-Gwon Kim, Young-Hoon Lee, Hye Seon Jeong, Lee Heeyoung, Won Kyung Lee, Seongheon Kim, Young-Kwon Park

**Affiliations:** 1 Department of Preventive Medicine, Gangwon Regional Cardiocerebrovascular Center, Kangwon National University Hospital, Chuncheon, Republic of Korea; 2 Department of Preventive Medicine, College of Medicine, Jeju National University, Jeju Regional Cardiocerebrovascular Center, Jeju National University Hospital, Aran 13gil 15, Jeju-si, Jeju, 63241 Republic of Korea; 3 Department of Preventive Medicine, School of Medicine, Kyungpook National University, Daegu, Republic of Korea; 4 Department of Health Policy and Management, School of Medicine, Jeju National University, Jeju, Republic of Korea; 5 Department of Health Informatics and Management, College of Medicine, Chungbuk National University, Cheong-ju, Republic of Korea; 6 Department of Preventive Medicine, School of Medicine, Chonnam National University, Gwangju, Republic of Korea; 7 Department of Neurology, Gyeongnam Regional Cerebrovascular Center, Gyeongsang National University Hospital, Jinju, Republic of Korea; 8 Busan Regional Cardiocerebrovascular Center, Dong-A University Hospital, Busan, Republic of Korea; 9 Department of Preventive Medicine, Wonkwang Medical Science, Wonkwang University School of Medicine, Jeonbuk Regional Cardiocerebrovascular Center, Wonkwang University Hospital, Iksan, Jeonbuk Republic of Korea; 10 Department of Neurology, Daejeon-Chungnam Regional Cardiocerebrovascular Center, Chungnam National University Hospital, Daejeon, Republic of Korea; 11 Seoul National University Bundang Hospital, Seongnam, Gyeonggi Republic of Korea; 12 Department of Prevention and Management, Inha University Hospital, School of Medicine, Inha University, Incheon, Republic of Korea; 13 Kangwon National University School of Medicine, Chuncheon, Republic of Korea; 14 Prevention and Management Center, Ulsan Regional Cardiocerebrovascular Center, Ulsan University Hospital, Ulsan, Republic of Korea

**Keywords:** Stroke, Disability, Quality of life, Medical adherence, Complications, Motivation, WHODAS 2.0

## Abstract

**Background:**

Little is known about the self-perceived level of disability of stroke survivors in the community. We aimed to characterise Health-related quality of life (HRQoL) 1 year after stroke and investigate how sociodemographic and stroke-related factors and medical adherence explain the self-perceived level of disability in a Korean stroke population.

**Methods:**

This was a multicentre cross-sectional study. A total of 382 ischaemic stroke survivors at 1 year after onset from 11 university hospitals underwent a one-session assessment, including socioeconomic variables, the modified Rankin Scale (mRS), various neurological sequelae, the Morisky, Green and Levin-Medication Adherence Questionnaire (MGL), and the World Health Organization Disability Assessment Schedule 2.0 (WHODAS 2.0) 36-items. The relationship between disability and different variables was analysed using ordinal logistic regression.

**Results:**

The prevalence of disability based on global WHODAS 2.0 was 62.6% (mild, 41.6%; moderate, 16.0%; severe, 5.0%). The prevalence of severe disability was higher in *participation in society* (16.8%) and *getting around* (11.8%) than in other domains. Low MGL- motivation was the only factor determining a significant association between all six domains of disability after adjustment. Different predictors for specific domains were age, mRS, dysarthria, trouble seeing, cognition problems, and MGL-motivation for *understanding and communicating*; age, recurrent stroke, mRS, hemiplegia, facial palsy, general weakness, and MGL-motivation for *getting around*; age, education, mRS, hemiplegia, and MGL-motivation for *self-care*; education, recurrent stroke, hemiplegia, dysarthria, and MGL-motivation for *getting along with people*; age, education, income, mRS, hemiplegia, dysarthria, MGL-knowledge, and MGL-motivation for *life activities*; living without a spouse, mRS, hemiplegia, dysarthria, trouble seeing, cognition problems, general weakness, and MGL-motivation for *participation in society*.

**Conclusions:**

Self-perceived disability according to the WHODAS 2.0 at 1 year after stroke was highly prevalent. Each disability domain showed a different prevalence and associated factors. Interventions promoting medical adherence to motivation seemed to help achieve high HRQoL in all domains.

**Supplementary Information:**

The online version contains supplementary material available at 10.1186/s12883-022-03032-2.

## Background

Stroke is a common and serious non-communicable health problem. It is the second leading cause of mortality [1] and the third leading cause of disability-adjusted life years [2]. In Korea, the Epidemiologic Research Council of the Korean Stroke Society reported an age- and sex-standardised incidence of first-ever stroke of 92.2 per 100,000 population in 2013, an age-standardised prevalence of stroke of 1.37% in Korean adults aged > 19 years in 2014, and an age-standardised stroke mortality of 29.6/100,000 population in 2015 [3]. Stroke was the third leading cause of disability-adjusted life years in Korea following diabetes mellitus and low back pain in 2012 [4].

Outcome assessment of acute stroke traditionally focuses on the prevention of death, alleviation of symptoms, impairments, and restoration of function [5]. However, health-related quality of life (HRQoL) measures may capture patients’ perceptions of disability better than clinicians’ assessment. This is not only because they are multidimensional instruments that comprise functional, physical, cognitive, psychological, and social elements [6], but also because the impact of limitations following a stroke on wellbeing may differ for each patient [7]. Furthermore, they reflect on health from their own perspectives [8].

World Health Organization Disability Assessment Schedule 2.0 (WHODAS 2.0) is a generic instrument of HRQoL for measuring function and disability in major life domains linked to the International Classification of Functioning, Disability, and Health (ICF). It is reliable and applicable across cultures of adult populations [9–12].

In Korea, the level of disability of stroke survivors in the community is unknown. We aimed to characterise HRQoL 1 year after stroke using WHODAS 2.0 and to investigate how sociodemographic factors, stroke-related factors, and medical adherence explain the self-perceived level of disability in a Korean stroke population.

## Methods

### Study design and population

This multicentre, cross-sectional study was conducted between December 2015 and March 2016. A total of 426 participants were recruited from the neurology outpatient clinics of 11 university hospitals designated as Regional Cardiocerebrovascular Centres (RCCs) in Korea (Daegu-Gyeongbuk, Gangwon, Jeju, Chungbuk, Gwangju-Jeonnam, Gyeongnam, Daejeon-Chungnam, Jeonbuk, Busan-Ulsan, Inchoen, and Gyeongi RCC) [13] and 382 respondents completed all assessments. Participants were stroke survivors who had been admitted to one of the RCC hospitals due to acute ischaemic stroke that occurred 12 to 15 months before the interview and who were willing to be informants. A one-on-one interview was conducted by trained nurses at 11 hospitals using a structured questionnaire. Patients who were unable to communicate independently were also excluded. Written informed consent was obtained from all the participants. The study protocol was approved by the Institutional Review Board of Kangwon National University Hospital.

### Measurement

#### Sociodemographic factors and stroke-related data

Data on sociodemographic and stroke-related characteristics were also collected (Additional file 1). The common sociodemographic variables on general characteristics are sex, age, living with a spouse or not, highest education qualification (elementary school/middle school/high school/college and above) and monthly household income (1 and less/1 to 2/more than 2 million Korea won; 1.2 million Korea won = 1000 USD). Stroke-related variables include recurrent or first-ever stroke, modified Rankin Score (mRS), and complications after stroke (hemiplegia, dysarthria, facial palsy, trouble seeing, paraesthesia, cognition problems, and general weakness) [14]. The mRS is robust and is the most commonly recommended functional measure in acute stroke research [5, 7, 14]. We categorised mRS into ‘normal to mild’ with a score ranging from 0 to 2 and ‘moderate to severe’, from 3 to 5 [15].

#### Self-reported medication adherence

The Morisky, Green and Levin-Medication Adherence Questionnaire (MGL) is a self-report measure of medication adherence. It was originally developed to predict the adherence of outpatients to antihypertensive medications using four items in the mid-1980s [16]. The MGL along with the two additional questions measure two domains of adherence (knowledge and motivation). Three items relating to knowledge were as follows. ‘When you feel better, do you sometimes stop taking your medicine’? ‘Sometimes, if you feel worse when you take your medicine, do you stop taking it’? ‘Do you know the long-term benefits of taking your medicine as told by your doctor or pharmacist’? The three relating to motivation are as follows. ‘Do you ever forget to take your medicine’? ‘Are you careless at times about taking your medicine’? and ‘Sometimes, do you forget to refill your prescription medicine on time’? Each item has a score of 0 or 1; a higher score indicates high adherence. MGL score can be categorised into ‘low’ with a score ranging from 0 to 1 and ‘high’ from 2 to 3 for each subdomain [17].

#### Health-related quality of life (HRQoL)

We measured HRQoL at 12 to 15 months post-ischaemic stroke using the WHODAS 2.0, a standardised cross-cultural measurement of disability [9]. The WHODAS 2.0 questionnaire has several forms according to the number of items, administration, and respondents. We used the WHODAS 2.0, 36-items covering six domains of functioning: *understanding and communicating* (six items), *getting around* (five items), *self-care* (four items), *getting along with people* (five items), *life activities* (eight items), and *participation in society* (eight items) [10]. We computed six domain-specific scores using 36-item complex scoring. The score ranges from 0 to 100, with a higher score indicating greater disability, such as lower QoL [10]. WHODAS 2.0 domain-specific and global scores were originally categorised into five grades: no problem (0–4%), mild disability (5–24%), moderate disability (25–49%), severe disability (50–95%), and extreme disability (96–100%). There were few subjects with extreme disability in this study; therefore, the five groups were collapsed into 4: no, mild, moderate, and severe disability. The reliability and validity of the Korean version have been established [18].

### Statistical analysis

We analysed data from 382 participants who completed all assessments. For descriptive purposes, absolute numbers and percentages were calculated for categorical variables and mean ± standard deviation (SDs) for continuous variables. The six domain-specific scores of WHODAS 2.0 were separately treated as dependent variables. Ordinal logistic regression was used since we had categorised for the dependent variable. The link function used for model fitting was the logit function. The overall model fit to the data was evaluated using model fitting information. A model exhibits a good fit to the data when a significant improvement in the fit of the final model contains a full set of independent variables over the null model. A parallel line test confirmed that the proportional odds assumption was satisfied for every ordinal logistic regression model. Every estimated ordinal logistic regression coefficient was transformed into an odds ratio, as the exponential of a particular coefficient was an estimate of the odds ratio. Data analyses were performed using SPSS version 24.0 (SPSS Inc., Chicago, IL, USA), and *p*-value less than 0.05 was considered statistically significant.

## Results

### Participants’ characteristics

The general characteristics of the 382 participants are summarised in Table 1. The participants’ mean age was 65.7 ± 12.2 years. Most participants (272, 71.2%) lived with their spouses. Additionally, 148 (38.7%) had a monthly family income of less than 1000,000 Korean won (2,660,000 Korean won was the median income for a two-person household in 2015) [19]. The details of stroke-related characteristics and medication adherence of the participants are listed in Table 2. Overall, 332 (86.9%) patients had normal to moderate levels of mRS, and hemiplegia (129, 33.8%) and dysarthria (92, 24.1%) were the most frequent complications. The level of self-reported medication adherence in the MGL-knowledge (370, 96.9%) was higher than that in the MGL-motivation (331, 86.6%).

**Table 1:** General characteristics of the participants (*N* = 382)

Characteristics	N (%)
Age	65.7 ± 12.2 years
Sex: Female	138 (36.1%)
Living with spouse	272 (71.2%)
Highest academic qualification	
Elementary school	144 (37.7%)
Middle school	68 (17.8%)
High school	105 (27.5%)
College and above	65 (17.0%)
Monthly family income (Korean won)^a^	
1,000,000 and less	148 (38.7%)
More than 1,000,000 to 2,000,000	93 (24.3%)
More than 2,000,000	141 (36.9%)

^a^1.2 million Korean won ≒1000 USD

**Table 2:** Stroke-related characteristics and medication adherence of the participants

Characteristics	N (%)
First-ever stroke	319 (83.5%)
mRS^a^	
Normal to mild	332 (86.9%)
Moderate to severe	50 (13.1%)
Complication after stroke	
Hemiplegia	129 (33.8%)
Dysarthria	92 (24.1%)
Facial palsy	13 (3.4%)
Trouble seeing	17 (4.5%)
Paresthesia	13 (3.4%)
Cognition problem	10 (2.6%)
General weakness	15 (3.9%)
MGL knowledge	
Low	12 (3.1%)
High	370 (96.9%)
MGL motivation	
Low	51 (13.4%)
High	331 (86.6%)

*mRS* modified Rankin Score, *MGL* Morisky, Green and Levin-Medication Adherence Questionnaire

^a^Normal to mild mRS < 3; moderate to severe mRS ≥ 3

### Domain-specific levels of WHODAS and associated factors

**Fig. 1:**
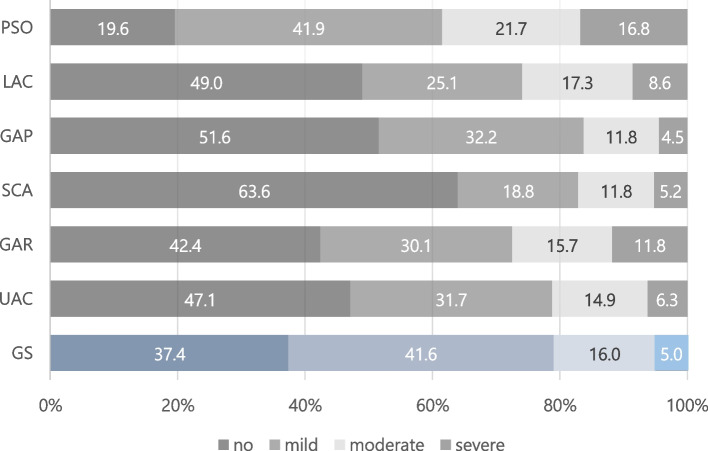
Distribution of WHODAS global scores by domain. GS = Global scores; UAC = Understanding and communicating; GAR = Getting around; SCA = Self-care; GAP = Getting along with people; LAC = Life activities; PSO = Participation in society

Among the 382 participants, the prevalence based on the WHODAS 2.0 level was 37.4% for no (disability-free), 41.6% for mild, 16.0% for moderate, and 5.0% for severe disability in Global scores. People with no disability were relatively common in *self-care* (63.6%) and *getting along with people* (51.6%). In contrast, the prevalence of severe disability was higher in *participation in society* (16.8%) and *getting around* (11.8%) than in the other domains of the WHODAS 2.0 (Fig. 1).

Table 3 shows the associations between different variables and disabilities in the domain-specific WHODAS 2.0 scores. The adjusted odds ratios (aORs) obtained from ordinal logistic regression models for different variables represent disability in the index group compared to those in the reference group. The results for domain-specific scores were adjusted for five demographic, nine stroke-related, and two medication adherence variables.

**Table 3:** Association between WHODAS 2.0 domains and selected variables

Variable	Understanding and Communicating			Getting Around			Self-Care		
	aOR	(95% CI)	*P*	aOR	(95% CI)	*P*	aOR	(95% CI)	*P*
Age (years)	**1.03**	**(1.01–1.05)**^a^	**0.010**	**1.06**	**(1.04–1.09)**	**< 0.001**	**1.07**	**(1.04–1.10)**	**< 0.001**
Sex: female	1.21	(0.77–1.90)	0.404	1.25	(0.79–1.97)	0.339	0.86	(0.50–1.48)	0.582
Living without spouse	0.97	(0.61–1.56)	0.915	1.05	(0.66–1.69)	0.826	1.44	(0.84–2.47)	0.181
Highest academic qualification									
Elementary school	1.44	(0.68–3.06)	0.344	0.80	(0.38–1.70)	0.565	1.30	(0.50–3.34)	0.590
Middle school	2.07	(0.96–4.45)	0.063	0.91	(0.42–1.94)	0.799	**2.66**	**(1.02–6.92)**	**0.045**
High school	1.32	(0.66–2.63)	0.434	0.84	(0.43–1.66)	0.617	**2.92**	**(1.20–7.12)**	**0.018**
College and above		1			1			1	
Monthly family income (10,000won)									
100 and less	0.73	(0.43–1.24)	0.243	1.10	(0.63–1.87)	0.733	1.58	(0.86–2.93)	0.143
100 to 200	1.30	(0.77–2.18)	0.039	1.10	(0.65–1.86)	0.996	1.25	(0.69–2.30)	0.479
More than 200		1			1			1	
Recurrent stroke	1.22	(0.71–2.11)	0.465	**1.88**	**(1.08–3.26)**	**0.024**	1.42	(0.78–2.59)	0.253
mRS^b^									
Normal to mild		1			1			1	
Moderate to severe	**4.04**	**(2.06–7.93)**	**< 0.001**	**8.27**	**(4.03–16.96)**	**< 0.001**	**11.60**	**(5.50–24.46)**	**< 0.001**
Complication after stroke									
Hemiplegia	1.41	(0.87–2.28)	0.159	**3.86**	**(2.37–6.27)**	**< 0.001**	**5.32**	**(3.06–9.28)**	**< 0.001**
Dysarthria	**1.88**	**(1.17–3.03)**	**0.010**	1.32	(0.81–2.15)	0.263	1.16	(0.67–1.98)	0.599
Facial palsy	2.76	(0.95–8.02)	0.063	**4.85**	**(1.55–15.21)**	**0.007**	1.70	(0.53–5.42)	0.372
Trouble seeing	**2.86**	**(1.11–7.38)**	**0.030**	1.44	(0.52–4.00)	0.480	1.97	(0.66–5.84)	0.222
Paresthesia	0.86	(0.26–2.79)	0.800	2.31	(0.76–7.02)	0.140	2.00	(0.48–8.23)	0.339
Cognition problem	**5.59**	**(1.61–19.38)**	**0.007**	0.65	(0.18–2.32)	0.506	1.30	(0.33–5.15)	0.706
General weakness	2.46	(0.88–6.88)	0.085	**3.19**	**(1.14–8.93)**	**0.027**	0.98	(0.24–3.93)	0.972
MGL knowledge^c^									
Low	2.15	(0.68–6.75)	0.191	2.24	(0.72–7.00)	0.164	2.93	(0.87–9.86)	0.082
High		1			1			1	
MGL motivation^c^									
Low	**3.12**	**(1.75–5.55)**	**< 0.001**	**3.22**	**(1.78–5.80)**	**< 0.001**	**2.88**	**(1.52–5.46)**	**0.001**
High		1			1	.		1	
Variable	Getting Along with People			Life Activities			Participation in Society		
	aOR	(95% CI)	P	aOR	(95% CI)	P	aOR	(95% CI)	P
Age (years)	1.02	(1.00–1.04)	0.051	**1.05**	**(1.03–1.07)**	**< 0.001**	1.00	(0.98–1.02)	0.979
Sex: female	1.34	(0.85–2.11)	0.208	1.42	(0.89–2.29)	0.146	1.13	(0.73–1.76)	0.589
Living without spouse	0.70	(0.43–1.13)	0.141	1.04	(0.64–1.70)	0.871	**1.76**	**(1.11–2.80)**	**0.017**
Highest academic qualification									
Elementary school	1.59	(0.74–3.40)	0.235	1.28	(0.57–2.86)	0.543	1.07	(0.52–2.21)	0.846
Middle school	**2.17**	**(1.01–4.67)**	**0.048**	**2.23**	**(1.00–5.01)**	**0.051**	1.24	(0.60–2.58)	0.565
High school	1.58	(0.80–3.14)	0.188	1.70	(0.82–3.54)	0.155	1.22	(0.65–2.30)	0.538
College and above		1			1			1	
Monthly family income (10,000won)									
100 and less	1.34	(0.78–2.29)	0.292	**1.77**	**(1.01–3.11)**	**0.048**	1.16	(0.69–1.97)	0.574
100 to 200	1.20	(0.70–2.04)	0.694	1.70	(0.98–2.96)	0.899	1.08	(0.64–1.83)	0.799
More than 200		1			1			1	
Recurrent stroke	**1.72**	**(1.00–2.94)**	**0.049**	1.30	(0.74–2.28)	0.367	1.73	(1.00–3.01)	0.050
mRS^b^									
Normal to mild		1			1			1	
Moderate to severe	1.03	(0.53–2.00)	0.924	**10.17**	**(4.84–21.35)**	**< 0.001**	**12.48**	**(5.77–27.00)**	**< 0.001**
Complication after stroke									
Hemiplegia	**2.72**	**(1.68–4.42)**	**< 0.001**	**6.23**	**(3.74–10.38)**	**< 0.001**	**3.87**	**(2.38–6.28)**	**< 0.001**
Dysarthria	**1.82**	**(1.13–2.94)**	**0.015**	**1.87**	**(1.14–3.06)**	**0.013**	**1.94**	**(1.19–3.18)**	**0.008**
Facial palsy	1.56	(0.53–4.62)	0.418	2.36	(0.77–7.20)	0.131	1.94	(0.62–6.04)	0.253
Trouble seeing	1.61	(0.61–4.26)	0.334	2.67	(0.97–7.39)	0.058	**5.45**	**(1.93–15.41)**	**0.001**
Paresthesia	1.51	(0.48–4.78)	0.485	1.17	(0.32–4.25)	0.810	1.53	(0.51–4.58)	0.447
Cognition problem	1.93	(0.56–6.63)	0.298	2.68	(0.77–9.31)	0.122	**6.06**	**(1.62–22.58)**	**0.007**
General weakness	2.28	(0.81–6.43)	0.120	1.19	(0.37–3.84)	0.777	**3.88**	**(1.38–10.92)**	**0.010**
MGL knowledge									
Low	2.23	(0.72–6.93)	0.164	**4.35**	**(1.31–14.44)**	**0.016**	2.19	(0.65–7.37)	0.207
High		1			1			1	
MGL motivation									
Low	**3.83**	**(2.16–6.82)**	**< 0.001**	**3.21**	**(1.76–5.82)**	**< 0.001**	**2.59**	**(1.42–4.70)**	**0.002**
High		1	.		1	.		1	

*mRS* modified Rankin Score, *MGL* Morisky, Green and Levin-Medication Adherence Questionnaire

^a^Adjusted odds ratios (95% confidence interval) from ordinal logistic regression models were estimated using age, sex, living without spouse, education level, family income level, recurrent stroke, mRS, hemiplegia, dysarthria, facial palsy, trouble seeing, paresthesia, cognition problem, general weakness, MGL knowledge and MGL motivation

^b^Normal to mild mRS < 3; moderate to severe mRS ≥ 3

^c^Low MGL knowledge (or motivation) < 2; high MGL knowledge (or motivation) ≥ 2

## Discussion

To our knowledge, this is the first detailed nationwide disability prevalence survey of ischaemic stroke patients at 1 year after onset in Korea. The study showed that the prevalence of disability based on the WHODAS 2.0 is 62.6%, almost double that of hemiplegia (33.8%), one of the most common neurological sequelae 1 year after stroke. The prevalence of severe disability (WHODAS 2.0, 50–100%) was higher in *participation in society* (16.8%) and *getting around* (11.8%) than in the other domains. The breakdown by domain also showed that prevalence decreased with severity. It also demonstrated that each domain of disability increases with various associated factors. In particular, age, recurrent stroke, moderate-to-severe mRS, hemiplegia, and dysarthria are generally related to different domains of disability, and low MGL- motivation is the only modifiable factor determining the significant association between all six domains of disability after adjustment.

Concerning personal background, age was associated with disability as in previous studies using WHODAS 2.0 [20–22]. This study indicated that older participants were more likely to have a greater disability in *understanding and communicating, getting around, self-care, and life activities*. Disability tends to increase with age. Older adults are more vulnerable to age-related comorbidities related to physical health problems [23]. However, even though the adjusted odds of being in a higher category in each domain except *self-care* was higher (aOR of 1.13 ~ 1.42) for females than males, these sex-related differences in WHODAS 2.0 disability measurements were not significant. A Korean study previously reported that older male stroke patients seem to be more vulnerable to self-care because of the Korean tradition of the passive domestic role of males [21]. The Framingham study reported that females with ischaemic stroke are not functionally more disabled than males [23].

A prior study considered *participation in society* as the most problematic and important because this domain involves the use of complex skills and navigation in daily life [22]. *Participation in society* is particularly limited by almost all the variables, such as living without a spouse, recurrent stroke, moderate to severe mRS, hemiplegia, dysarthria, trouble seeing, cognitive problems, general weakness, and low MGL- motivation. However, among the seven variables of neurological sequelae, both *life activities* and *getting along with people* are associated only with hemiplegia and dysarthria. This indicates *that participation in society* is not only about getting along with people but also about daily life.

Each neurological sequela was associated with different domains of WHODAS 2.0. For example, hemiplegia is associated with five domains: *understanding and communicating*, dysarthria with *understanding and communicating*, *getting along with people*, *life activities*, and *participation in society*, trouble seeing with *understanding and communicating* and *participation in society*, and general weakness with *getting around* and *participation in society*. Therefore, it is necessary to consider customised support, for example, a home visit to hemiplegic patients, which requires comprehensive services, or a going out companion to general weakness, which requires simpler services. It would be reasonable to manage these supports according to periodically assessed HRQoL.

It is of interest and importance that low MGL-motivation was significantly associated with all six domains of disability after adjustment (aOR of 2.59 ~ 3.83). Therefore, it would be worthwhile to improve the level of this modifiable variable. Medication adherence is usually defined as the proportion of days covered (PDC), the percentage of medication actually taken at the prescribed doses [24], at 1 year after stroke. The Epidemiologic Research Council of the Korean Stroke Society reported a much lower adherence compared to a previous study from the US [25] (75% vs. 91% for lipid-lowering drugs, 74% vs. 91% for antidiabetic drugs, and 82% vs. 92% for antihypertensive drugs) [3]. Moreover, unlike MGL-knowledge, MGL- motivation is associated with adherence to lifestyle modifications for risk reduction [26]. Such evidence implies that there is substantial room for improvement in the HRQoL of stroke survivors. It is necessary for stroke survivors to provide interventions to improve MGL- motivation using specific methods, such as tailored education, computer-based education, and mobile phone reminders.

This study had several limitations. Our participants are regarded as persons of higher socioeconomic status in the Korean context; the affluent likely have regular outpatient follow-ups at a particular university hospital. Thus, it is possible that overall, participants demonstrated mild deficits as well as a better level of adherence to their medication compared with stroke survivors in the general population. There is also a possibility of selection bias by excluding stroke survivors 1 year after the event due to difficulties in the interview, even though we tried to ensure that stroke survivors were eligible for the study. However, although this study included only ischaemic stroke, generalisation to haemorrhagic stroke is also possible.

In addition, the WHODAS 2.0 mainly covers the activities and participation domains of the ICF, so there has been a need to address bodily impairments and environmental factors [9]. However, this study chose several factors related to bodily impairments such as hemiplegia, dysarthria, and facial palsy. In future studies, environmental factors such as physical, attitudinal, and social barriers can be considered as other factors to determine disability better.

Finally, cognition problems and general weakness were under-reported and under-screened. In general, in Korea, these problems have not necessarily been assessed during outpatient clinics after 1 year of stroke. As neurologists have regarded these problems as non-specific symptoms which might have many possible causes for stroke survivors, they have started to pay special attention to the severity and cause only when patients mention these two complaints. For that reason, in this study as well, the frequency of these problems could be known by asking an open question what kind of discomfort you are currently experiencing due to the complications of stroke.

## Conclusions

Self-perceived disability by the WHODAS 2.0 was so highly prevalent that it had almost double the prevalence compared to hemiplegia, one of the most common neurological sequelae 1 year after stroke. Each domain of disability showed a different prevalence, which increased with various associated factors. Interventions promoting medical adherence to motivation seemed to help achieve high HRQoL in all domains.

## Supplementary Information

**Additional file 1.**

## Data Availability

The datasets generated and/or analysed during the current study are available from the corresponding author on reasonable request.

## Abbreviations

aORs: Adjusted odds ratios
HRQoL: Health-related quality of life
ICF: International Classification of Functioning, Disability, and Health
MGL: Morisky, Green, Levine-Medication Adherence Questionnaire
mRS: Modified Rankin Scale
PDC: Proportion of days covered
RCCs: Regional Cardiocerebrovascular Centres
SD: Standard deviation
USD: U.S.dollar
WHODAS 2.0: World Health Organization Disability Assessment Schedule 2.0
