# Supplementary material for: Health-related quality of life using WHODAS 2.0 and associated factors 1 year after stroke in Korea: a multi-centre and cross-sectional study
Source: BMC Neurol. 2022 Dec 24;22:501. doi: 10.1186/s12883-022-03032-2 (PMC9789571; doi:10.1186/s12883-022-03032-2)
Supplement: Supplementary file 1 — Additional file 1. [file 12883_2022_3032_MOESM1_ESM.docx]

| ***Sociodemographic factors and stroke-related data*** |
| --- |

1. Is there anything uncomfortable about your body right now due to the sequelae of the stroke?

① hemiplegia ② dysarthria ③ facial palsy ④ trouble seeing

⑤ Please write down anything else_________________________________________________

2. What is your final education?

① elementary school ② middle school ③ high school ④ college and above

3. What is the monthly income of all the household members living in the same house together?

① 1 and less million Korea won ② 1 to 2 million Korea won ③ more than 2 million Korea won

4. What is your current marital status?

① not married yet ② married (living together) ③ separated ④ divorced ⑤ died

5. Sex: ① M ② F

6. How old are you? I am _____________ years old now.

7. When did the first stroke occur? Year: _____________, month: _____________

8. How many strokes have you had so far? _____________

9. What is the current level of mRS? _____________
